# Early postoperative hemodynamic instability after heart transplantation – incidence and metabolic indicators

**DOI:** 10.1186/s12871-021-01455-x

**Published:** 2021-10-02

**Authors:** Anna Kędziora, Jacek Piątek, Hubert Hymczak, Grzegorz Wasilewski, Bartłomiej Guzik, Rafał Drwiła, Bogusław Kapelak, Dorota Sobczyk, Janusz Konstanty-Kalandyk, Karol Wierzbicki

**Affiliations:** 1 Department of Cardiovascular Surgery and Transplantology, John Paul II Hospital, 80 Pradnicka St., 31-202 Krakow, Poland; 2 Jagiellonian University Medical College, Krakow, Poland; 3 Department of Anesthesiology and Intensive Care, John Paul II Hospital, Krakow, Poland; 4 Faculty of Medicine and Health Sciences, Andrzej Frycz Modrzewski Krakow University, Krakow, Poland; 5 Department of Interventional Cardiology, John Paul II Hospital, Krakow, Poland; 6 Department of Cardiac and Vascular Diseases, John Paul II Hospital, Kraków, Poland

**Keywords:** Heart transplantation, Primary graft failure, Hemodynamic instability, Serum lactate, Vasoplegia, Inotrope score

## Abstract

**Background:**

Primary graft dysfunction (PGD) is the main cause of death in the first 30 days after heart transplantation (HTX), accounting for approximately 40% of mortality. The study’s primary aim was to assess the incidence of PGD, following the International Society for Heart and Lung Transplantation consensus, and to compare it with the incidence of significant postoperative hypotension despite administration of high-dose inotropes and vasoconstrictors. The secondary aim of the study was to determine changes in biochemical markers that accompany the phenomenon.

**Methods:**

Forty-five patients who underwent HTX between 2010 and 2015 were enrolled in this study, and detailed hemodynamic and metabolic data from the first 48 postoperative hours were collected and analyzed. Hemodynamic instability was defined as significant postoperative hypotension (mean arterial pressure (MAP) < 60 mmHg) combined with a high inotrope score (> 10). Data for long-term mortality were obtained from the population registration office.

**Results:**

PGD incidence was relatively low (17.8%); however, hemodynamic instability was common (40%). Among unstable patients, MAP was insufficient for end-organ perfusion (51.4 ± 9.5 mmHg) but no decrease in left ventricular function was observed (cardiac index, 2.65 ± 0.6 l/min/m2; left ventricular ejection fraction, 52.9 ± 15.5%). Within this group, mean systemic vascular resistance index (961 ± 288 dyn*s*m2/cm5) was low despite receiving high doses of vasoactive agent (norepinephrine 0.21 (0.06-0.27) μg/kg/min during first 24 h postoperatively and 0.21 (0.01-0.27) μg/kg/min during next 24 h postoperatively). After HTX, serum lactate levels were initially significantly higher in patients with hemodynamic instability (*p* = 0.002); however, impaired lactate clearance was not observed (*p* = 0.366), and lactate levels normalized within the first 24 h postoperatively. Postoperative hemodynamic instability altered the long-term outcome and increased 5-year mortality after HTX (*p* = 0.034).

**Conclusions:**

Hemodynamic instability is a more common phenomenon than PGD. Only early postoperative serum lactate levels correspond with hemodynamic instability following HTX. Postoperative hemodynamic instability is associated with poor long-term survival among HTX recipients.

## Background

Recent technical developments have allowed application of novel treatment strategies for heart failure (HF) management. Left ventricular assist devices (LVADs) are currently implanted worldwide as a destination therapy in over 70% of cases with excellent 3-year survival for centrifugal flow devices with full magnetic levitation [1]. However, in accordance with the current European Society of Cardiology (ESC) guidelines, heart transplantation (HTX) remains the gold standard treatment option that significantly improves survival, exercise capacity, and quality of life [2]. Apart from donor shortage, the early postoperative period remains the most challenging, with the highest mortality rate of 10–15%, steady throughout all transplant eras [3].

Primary graft dysfunction (PGD) is the main cause of death in the first 30 days after HTX, accounting for approximately 40% of mortality [3]. PGD incidence significantly varies across studies (from 2.3 to 28.2%), which represents a wide range of applied definitions, even after the International Society for Heart and Lung Transplantation (ISHLT) consensus was introduced in 2014 [4]. The consensus streamlined the diagnosis of a rigorously defined impaired cardiac function and the extent of inotrope and mechanical support to be required within 24 h of completion of surgery [4]. Since then, only a few papers have focused on the accurately reported incidence for PGD [5–7]. Nevertheless, the initial authors hypothesis was that postoperative hemodynamic complications may be a more common phenomenon that PGD. Therefore, the primary aim of the study was to assess the incidence of PGD in accordance with the ISHLT consensus and to compare it with the incidence of significant postoperative hypotension despite administration of high-dose inotropes and vasoconstrictors within the same sample.

The reported outcomes of severe PGD cases were usually fatal, and the most common autopsy findings included reperfusion injury, ischemia, and myocyte necrosis [4]. Serum lactate levels are an indicator of tissue ischemia in many clinical settings [8]. However, postoperative hyperlactatemia is commonly observed after HTX, and levels ≥4 mmol/L were reported in 59.2% of recipients in one study [9]. Based on our previous findings, serum lactate levels taken immediately after HTX can be used to predict in-hospital mortality [10, 11]. On the other hand, another study showed that extremely high lactate levels (severe hyperlactatemia defined by the authors as > 15 mmol/L) at any time of hospitalization was associated with fatal outcomes [12]. Nevertheless, a comprehensive analysis of postoperative serum lactate levels and hemodynamic function in HTX recipients is still lacking. Therefore, the secondary aim of the study was to determine the association between lactate levels and other routinely measured biochemical markers with hemodynamic parameters within early period after HTX.

## Material and methods

Forty-five patients who underwent HTX in the Department of Cardiovascular Surgery and Transplantology between 2010 and 2015 were enrolled in this retrospective study (Table 1). None of the patients were supported with LVAD prior to HTX. Detailed data were retrieved from medical records for the first 48 h after HTX. Data for long-term mortality were obtained from the population registration office.

**Table 1:** Baseline patients characteristic

	ALL HTX RECIPIENTS	WITH HEMODYNAMIC INSTABILITY	WITHOUT HEMODYNAMIC INSTABILITY	***p***-value
	***n = 45***	***n = 18***	***n = 27***	
**Demography**				
Sex				1.000
Female	4 (8.89)	2 (11.1)	2 (7.41)	
Male	41 (91.1)	16 (88.9)	25 (92.6)	
Age, years	48.5 (11.7)	51.2 (12.0)	46.7 (11.5)	0.215
BMI, kg/m^2^	25.6 (4.11)	27.9 (3.61)	24.3 (3.83)	0.009
Hiperlipidemia	15 (33.3)	6 (33.3)	9 (33.3)	1.000
Diabetes	11 (24.4)	7 (38.9)	4 (14.8)	0.086
Cardiomyopathy				0.732
Dilated	34 (75.6)	13 (72.2)	21 (77.8)	
Ischemic	11 (24.4)	5 (27.8)	6 (22.2)	
**Preoperative parameters**				
Urgent HTX	36 (80.0)	13 (72.2)	23 (85.2)	0.449
iv DB^a^	33 (73.3)	11 (61.1)	22 (81.5)	0.175
iv inotropic agents^b^	11 (24.4)	6 (33.3)	5 (18.5)	0.304
AST, U/L	29.0 (22.3)	23.6 (9.96)	32.4 (27.1)	0.132
ALT, U/L	32.1 (33.3)	27.2 (20.1)	35.2 (39.6)	0.382
TBIL, μmol/L	13.2 (8.86)	12.8 (7.59)	13.4 (9.75)	0.833
CREA, μmol/L	97.7 (29.1)	114 (32.1)	87.1 (21.4)	0.005
GFR, mL/min/1.73m^2^	77.7 (20.5)	65.6 (18.3)	85.8 (17.9)	0.001

Number (%), mean (SD) are presented

*AST* aspartate aminotransferase, *ALT* alanine aminotransferase, *TBIL* total bilirubin, *CREA* creatinine, *GFR* glomerular filtration rate

^a^pretransplant continuous intravenous dobutamine infusion

^b^pretransplant continuous intravenous inotropic agents infusion (other than dobutamine)

PGD incidence and severity were assessed in accordance with the ISHLT consensus [4]. Hemodynamic instability was defined as any episode of mean arterial pressure (MAP) below 60 mmHg [8] combined with high-dose inotropes and vasoconstrictors requirement (inotrope score > 10) [4] or need for venoarterial extracorporeal membrane oxygenation (VA-ECMO).

### Intraoperative management

All surgeries were performed via median sternotomy using the biatrial technique. All recovered hearts were protected with crystalloid cardioplegia (Celsior®), as described previously [9]. Sufentanil (0.2 to 0.5 μg/kg) was administered for general anesthesia, followed by an induction agent (propofol 0.5 to 1 mg/kg or etomidate 0.2 to 0.4 mg/kg for unstable patients), and a non-depolarizing neuromuscular blocking agent. Arterial blood gas (ABG) analysis (including hemoglobin level, serum glucose, serum lactate, and serum potassium analyses) was performed before induction of general anesthesia, after initiation of cardiopulmonary bypass (CPB), during CPB, before weaning CPB, and after CPB. All metabolic abnormalities were corrected. Protective mechanical ventilation was carried out with 6-8 mL/kg predicted body weight with the optimal settings of positive end-expiratory pressure and rate to prevent hypoxia, hypercarbia, and increase in pulmonary vascular resistance. Inhaled nitric oxide was used if necessary. The hemodynamic status was continuously monitored and corrected with the lowest effective dose of intravenous inotropic or vasoactive agent infusion when necessary. Levosimendan was routinely used for all patients in the first 24 h after induction of anesthesia (0.1 μg/kg/min) without a loading dose.

### Postoperative management

Postoperative management included continuous ECG and invasive arterial blood pressure monitoring for systolic blood pressure, diastolic blood pressure, and MAP. A Swan-Ganz catheter was used for detailed hemodynamic measurements, including central venous pressure (CVP), pulmonary artery systolic pressure (PA), pulmonary capillary wedge pressure (PCWP), cardiac output (CO) and cardiac index (CI), systemic vascular resistance index (SVRI), and pulmonary vascular resistance index (PVRI). Detailed data were obtained every 6 h (Table 2). Transthoracic echocardiography (TTE) with left ventricle ejection fraction (LVEF) assessment was performed daily. Within the first 48 h, all patients received mycophenolate mofetil, corticosteroids, and anti-thymocyte globulin for immunosuppression induction. Calcineurin inhibitors were added in further treatment schemes. Continuous infusion of inotropic or vasoactive agents was used when necessary with the lowest effective dose and data were collected daily (Table 2). The inotrope score was established daily and calculations were made in accordance with the ISHLT consensus on PGD [4].

**Table 2:** Hemodynamic parameters and inotropic and vasoactive agents requirement from first 48 h after HTX

	ALL HTX RECIPIENTS	WITH HEMODYNAMIC INSTABILITY	WITHOUT HEMODYNAMIC INSTABILITY	***p***-value
	***N = 45***	***N = 18***	***N = 27***	
**Hemodynamic parameters**				
LVEF_min, %	54.4 (12.4)	52.9 (15.5)	55.4 (10.2)	0.572
CI_min, l/min/m2	2.54 (0.67)	2.65 (0.58)	2.48 (0.71)	0.405
MAP_min, mmHg	61.8 (10.8)	51.4 (9.49)	67.8 (5.75)	< 0.001
HR_max, bpm	103 (20.9)	102 (21.7)	104 (20.9)	0.765
SBP_min, mmHg	94.7 (12.7)	84.3 (11.0)	101 (9.43)	< 0.001
PA_max, mmHg	39.5 (7.01)	41.9 (6.00)	38.1 (7.29)	0.081
CVP_max, mmHg	15.4 (3.10)	16.3 (2.85)	14.8 (3.16)	0.132
PCWP_max, mmHg	16.8 (3.02)	18.2 (3.30)	16.0 (2.60)	0.039
SVRI_min, dyn*s*m2/cm5	1209 (369)	961 (288)	1328 (348)	0.002
**Inotropic and vasoactive agents**^**a**^				
0-24 h after HTX				
ADR, μg/kg/min	0.15 (0.01-0.17)	0.25 (0.05-0.30)	0.09 (0.01-0.11)	0.002
NA, μg/kg/min	0.12 (0.00-0.15)	0.21 (0.06-0.27)	0.06 (0.00-0.13)	0.007
DB, μg/kg/min	7.48 (4.36-10.16)	7.83 (3.45-11.67)	7.29 (4.34-9.92)	0.714
MIL, μg/kg/min	0.27 (0.11-0.36)	0.34 (0.20-0.43)	0.24 (0.08-0.34)	0.257
Inotrope score^b^	16.2 (9.8-41.4)	37.1 (14.7-79.8)	12.7 (9.2-30.9)	0.012
24-48 h after HTX				
ADR, μg/kg/min	0.12 (0.01-0.14)	0.26 (0.04-0.36)	0.03 (0.00-0.04)	0.000
NA, μg/kg/min	0.09 (0.01-0.11)	0.21 (0.01-0.27)	0.03 (0.00-0.05)	0.002
DB, μg/kg/min	7.30 (4.30-9.89)	7.64 (2.14-11.98)	7.13 (4.24-9.51)	0.955
MIL, μg/kg/min	0.24 (0.10-0.36)	0.30 (0.13-0.40)	0.20 (0.09-0.32)	0.120
Inotrope score^b^	13.3 (8.6-35.5)	35.1 (11.9-77.4)	11.3 (6.6-23.1)	0.011

Number (%), mean (SD) or median (Q1-Q3) are presented

*LVEF* left ventricle ejection fraction, *CI* cardiac index, *MAP* mean arterial pressure, *HR* heart rate, *SBP* systolic blood pressure, *PA* pulmonary artery systolic pressure, *CVP* central venous pressure, *PCWP* pulmonary capillary wedge pressure, *SVRI* systemic vascular resistance index, *ADR* epinephrine, *NA* norepinephrine, *DB* dobutamine, *MIL* milrinone

^a^ levosimendan was routinely used for all patients for first 24 h after induction of anesthesia (0.1 μg/kg/min)

^b^inotrope score calculated in accordance with ISHLT consensus^4^: (dopamine (×1) + dobutamine (×1) + amrinone (×1) + milrinone (× 15) + epinephrine (× 100) + norepinephrine (× 100) with each drug dosed in μg/kg/min)

ABG analysis was conducted at the necessary frequency (every 2 – 6 h), based on the patient’s clinical condition. Detailed data were obtained every 6 h in accordance with the established protocol [10]. Creatinine (CREA), glomerular filtration rate (GFR), total bilirubin (TBIL), alanine aminotransferase (ALT), and aspartate aminotransferase (AST) levels were monitored daily. GFR was automatically calculated by the medical analytics facility based on Modification of Diet in Renal Disease (MDRD) equation.

### Statistical analysis

Statistical analysis was performed using the R Statistical Software (Foundation for Statistical Computing, Vienna, Austria). Normal distribution was tested using the Shapiro-Wilk test. Continuous variables are presented as means and standard deviations (±SD) or medians and quartiles (Q1-Q3). For categorical variables, numbers and proportions were reported. Parametric and non-parametric tests, when appropriate, were used for either independent samples or repeated measurements. McNemar’s test was used to determine the difference between PGD and hemodynamic instability incidence. Repeated measures ANOVA and area under the curve analysis were used for comparison of biochemical measurements between groups. Univariate survival analysis for remodeling function was performed using Kaplan–Meier survival plots and log rank tests.

## Results

**Fig. 1:**
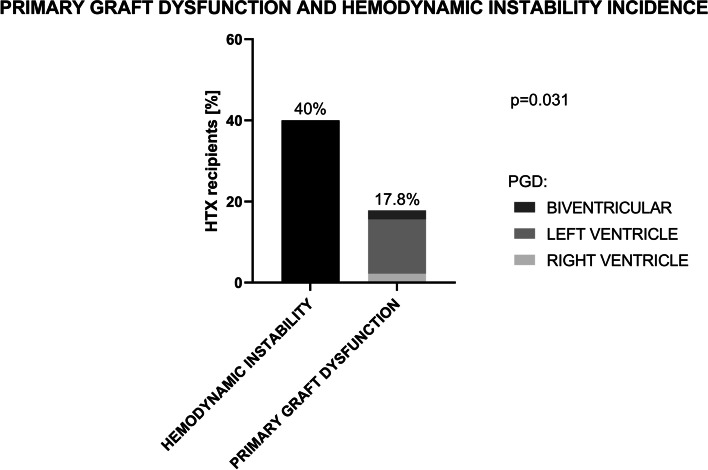
Primary graft dysfunction and hemodynamic instability incidence; *McNemar’s test*

PGD was identified in 8 (17.8%) cases and mostly involved the left ventricle (6 patients, 13.3%). In two cases (4.4%) PGD was severe and patients required VA-ECMO support. Hemodynamic instability was more common than PGD and was observed in 18 (40%) patients (Fig. 1; *p* = 0.031).

Among patients with hemodynamic instability left ventricle function was not impaired postoperatively (CI 2.65 ± 0.6 l/min/m^2^; LVEF 52.9 ± 15.5%) (Table 2); however, MAP was markedly low and insufficient for end-organ perfusion (51.4 ± 9.5 mmHg). Moreover, mean SVRI was significantly lower when compared to patients without hemodynamic instability (961 ± 288 vs 1328 ± 348 dyn*s*m^2^/cm^5;^ *p* = 0.002) despite administration of higher doses of norepinephrine and higher overall inotrope score (Table 2). Bacterial infection was ruled out in all of the patients based on laboratory tests results (leucocyte count, procalcitonin) obtained within analyzed period.

**Fig. 2:**
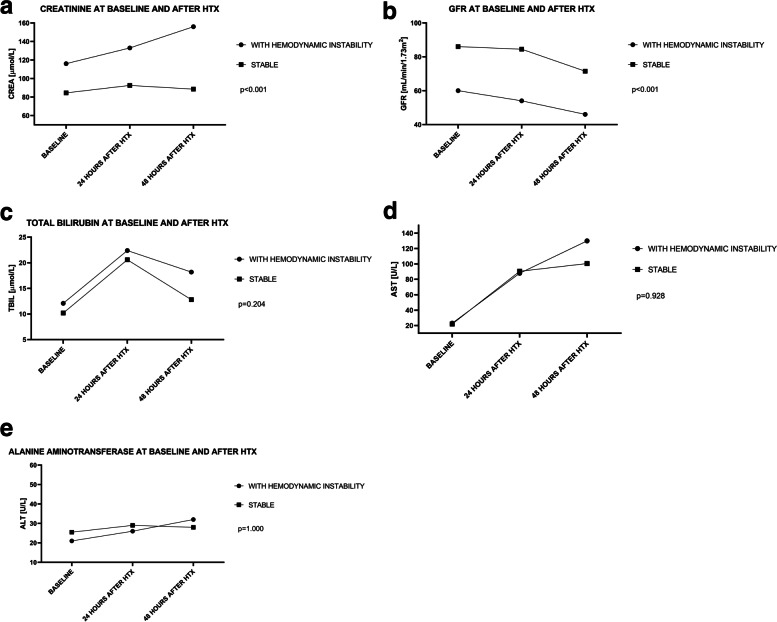
Area under the curve analysis for baseline and postoperative trends: **A** creatinine, **B** GFR, **C** total bilirubin, **D** aspartate aminotransferase, E. alanine aminotransferase; *median values are presented*

Postoperatively, patients with hemodynamic instability had higher and increasing creatinine levels (Fig. 2A); however, preexisting impaired renal function was observed in this group (Table 1). Postoperative liver function did not vary between the groups (Fig. 2C, D, E).

**Fig. 3:**
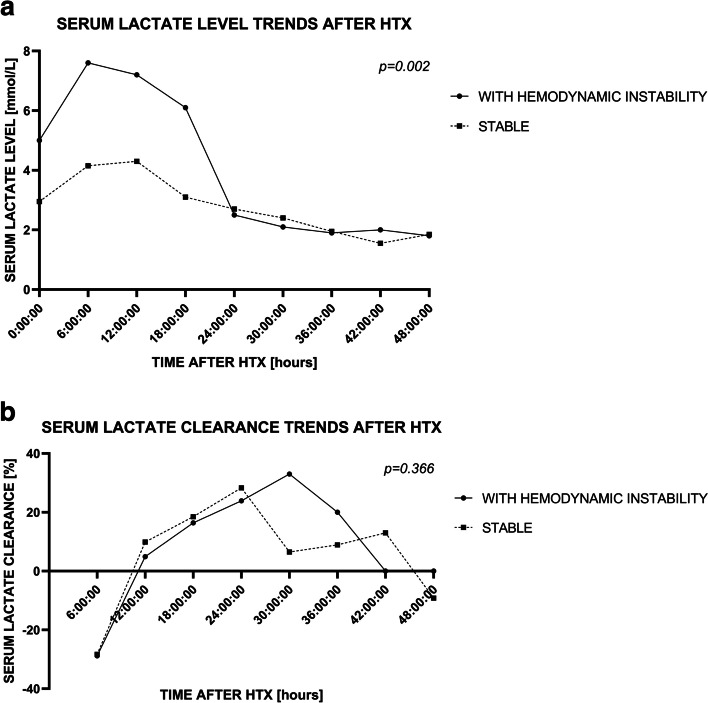
Area under the curve analysis for postoperative trends: **A** serum lactate level, **B** lactate clearance; *median values are presented*

On the other hand, postoperative serum lactate levels were significantly higher in recipients with hemodynamic instability (Fig. 3A; *p* = 0.002). Higher levels were observed among these patients upon intensive care unit admission (5.0 (4.0-7.2) vs 3.0 (1.7-6.2) mmol/L; *p* = 0.024) despite no significant difference in surgery time (320 (300-340) vs 270 (210-315) minutes; *p* = 0.118), CPB time (135 (122-202) vs 142 (128-195) minutes; *p* = 0.238), and total ischemic time (215 (141-225) vs 204 (185-245) minutes; *p* = 0.410). Moreover, difference in postoperative serum lactate levels was present only within first 24 h after HTX (Fig. 3A), despite high-dose inotropes requirement throughout all 48 h of observation (Table 2). Serum lactate levels did not differ between the groups at 24 h after HTX (2.5 (2.2-5.3) vs 2.7 (1.5-3.6); *p* = 0.174) and lactate clearance was surprisingly not impaired among HTX recipients with hemodynamic instability (Fig. 3B; *p* = 0.366).

**Fig. 4:**
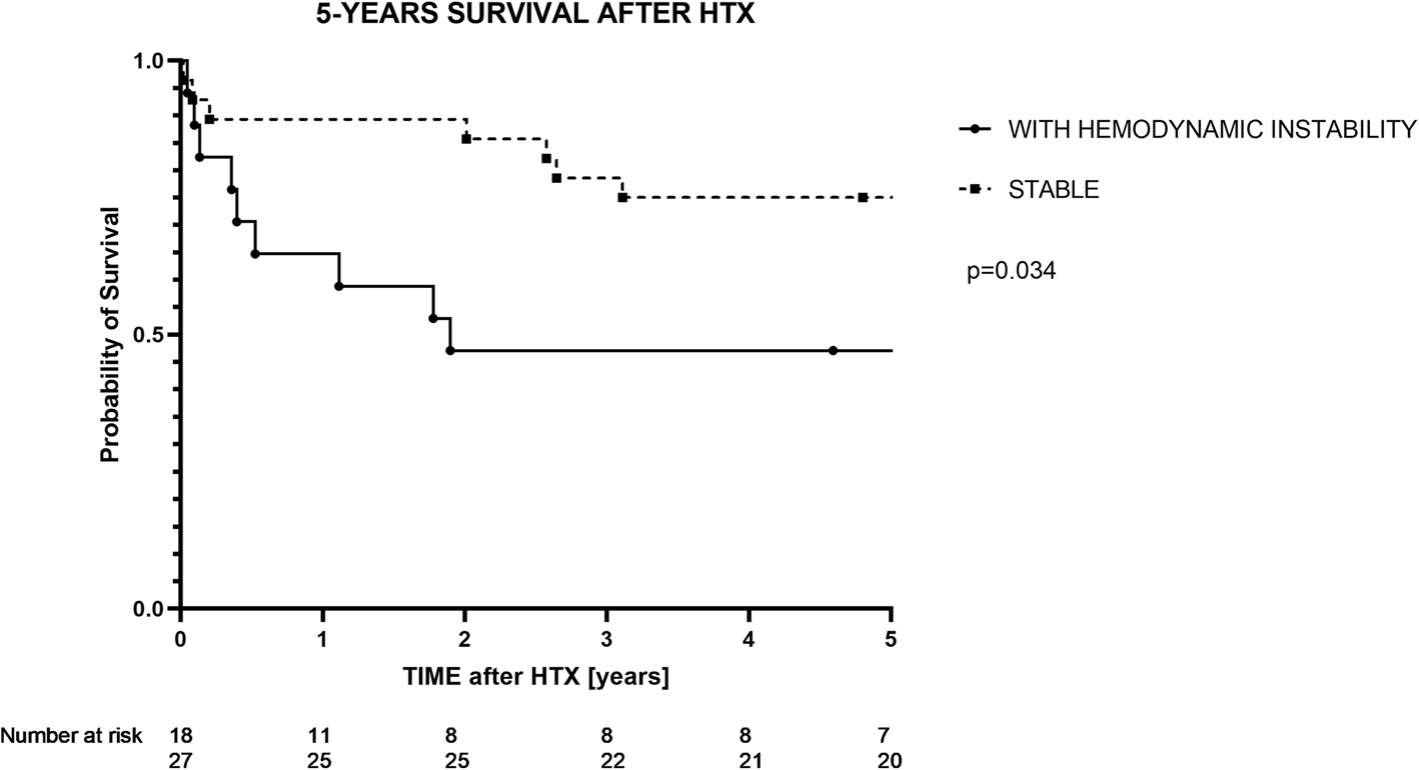
Kaplan-Meier 5-year survival analysis; *log-rank test*

Five in-hospital deaths were reported (11.1%), mostly at a distant postoperative day (median: 30 [12-43] days). Three out of five (60%) deceased patients had postoperative hemodynamic instability (60% vs 40%; *p* = 0.277). However, postoperative hemodynamic instability altered the long-term outcome and increased 5-years mortality (Fig. 4).

## Discussion

Two of the largest studies that followed the ISHLT diagnostic criteria for PGD reported an incidence of 36% in the UK [5] and 15.3% for severe PGD requiring mechanical circulatory support in the American population [7]. Thus, the total PGD incidence in this study was relatively low (17.8%) and venoatrial extracorporeal circulation was necessary only in 2 patients with severe left ventricle PGD (4.4%). However, hemodynamic instability was more common and observed in 40% of the cases. Similar to data on PGD, patients with hemodynamic instability had declined preoperative renal function and a higher incidence of diabetes [4, 11–13]. Moreover, patients with hemodynamic instability had higher BMI, which may have resulted in a higher risk of a donor-recipient size mismatch, a known parameter for poor prognosis after HTX [5]. Although the study was not designed to assess the preoperative risk factors for hemodynamic instability but to determine postoperative biochemical markers of the phenomenon, these important baseline differences cannot be overlooked.

On the contrary to PGD, acceptable CI and LVEF were reported among patients with hemodynamic instability, presumably due to high doses of inotropic support that were used within early postoperative period (Table 2). However, the observed insufficient for end-organ perfusion MAP combined with the low SVRI despite significantly higher vasoactive agent doses (Table 2) is an interesting finding that reflects a potential vasopressor-dependent distributive component in hemodynamics after HTX. The activation of the systemic inflammatory response in the recipient, resulting in a vasodilated systemic circulation, has been hypothesized as one of the important aspects of pathophysiology of PGD [4]. However, due to the non-uniform diagnostic criteria, the data on this topic are sparse and a high range of reported vasoplegia syndrome incidence after HTX have been noted (11-54%) in a few single center reports [14, 15].

Nevertheless, early and proper identification of patients with hemodynamic instability seems to be crucial, as the impact on overall long-term survival was shown in this study (Fig. 4). The long-term effect of PGD has been previously noted, and the increase in overall mortality was observed throughout the first 3 months after HTX in one paper [12]. This study showed that the impact of early postoperative hemodynamic status is even greater, and the significant decline of the survival curve can be seen up to 2 years after HTX (Fig. 4).

However, there is a major gap in scientific evidence regarding the early postoperative management of HTX recipients. Most of the recommendations arise from expert consensus and no unified strategy for biochemical parameters monitoring is established [16]. As shown in this study, serum lactate levels correlate with the hemodynamic status and measurements obtained within early hours after HTX may predict hemodynamic instability and higher requirement for inotropic and vasoactive agents (Fig. 3A). Therefore, early and strict monitoring of lactate levels provides an opportunity to identify patients at higher risk of developing hemodynamic instability and to implement corrective measures to improve the outcome.

Another finding worth emphasizing is the fact that in contrast to other clinical settings (i.e., liver transplantation, mitral valve surgery, sepsis) [17–19] lactate clearance was not impaired among unstable patients. The initial difference in serum lactate levels between the groups resolved within first 24 h of observation and serum lactate levels returned to normal ranges within the first 48 h after HTX. The cause of this unique phenomenon that distinguish HTX recipients from all previously analyzed patients hospitalized in the intensive care unit [17–19] cannot be established by this study results. The initial authors’ hypothesis was that significant decline in liver and renal function, as a result of need for increased lactate utilization, will be observed, however, obtained data cannot support such statement.

The retrospective study design may raise concerns regarding the quality of evidence; however, consecutive patients were enrolled, and no patient was excluded from the analysis. Despite good adherence to the institutional postoperative management protocol regarding the timing of repeated recipients’ assessment in the ICU, several anesthesiologists supervised the postoperative care within the early hours after HTX. Therefore, slight variations in decisions on fluid management and inotropic support might have been present, and investigators cannot calculate this effect in this study. Nevertheless, the major limitations of our study are the small sample size and single-center data. Study findings will require further validation in a higher volume population with a prospective design and collection of additional data on fluid management.

## Conclusions

Hemodynamic instability is a more common phenomenon than PGD. Only early postoperative serum lactate levels correspond with hemodynamic parameters following HTX as impaired lactate clearance is not observed among patients with hemodynamic instability. Postoperative hemodynamic instability is associated with poor long-term survival among HTX recipients.

## Data Availability

The datasets used and analyzed during the current study are available from the corresponding author on reasonable request.

## Abbreviations

PGD: Primary Graft Dysfunction
HTX: Heart transplantation
MAP: Mean arterial pressure
HF: Heart failure
LVAD: Left ventricular assist device
ESC: European Society of Cardiology
ISHLT: International Society for Heart and Lung Transplantation
VA-ECMO: Venoarterial extracorporeal membrane oxygenation
ABG: Arterial blood gas
CPB: Cardiopulmonary bypass
ECG: Electrocardiography
CVP: Central venous pressure
PA: Pulmonary artery systolic pressure
PCWP: Pulmonary capillary wedge pressure
CO: Cardiac output
CI: Cardiac index
SVRI: Systemic vascular resistance index
PVRI: Pulmonary vascular resistance index
TTE: Transthoracic echocardiography
LVEF: Left ventricle ejection fraction
CREA: Creatinine
GFR: Glomerular filtration rate
TBIL: Total bilirubin
ALT: Alanine aminotransferase
AST: Aspartate aminotransferase
